# Metformin inhibits ALK1-mediated angiogenesis via activation of AMPK

**DOI:** 10.18632/oncotarget.15825

**Published:** 2017-03-02

**Authors:** Ying Ying, Takashi Ueta, Shanshan Jiang, Hui Lin, Yuanyuan Wang, Demetrios Vavvas, Rong Wen, Ye-Guang Chen, Zhijun Luo

**Affiliations:** ^1^ Jiangxi Province Key Laboratory of Tumor Pathogens and Molecular Pathology, Department of Pathology, Schools of Basic Medical Sciences and Pharmaceutical Sciences, Nanchang University Medical College, Nanchang, China; ^2^ Department of Biochemistry, Boston University School of Medicine, Boston, MA, USA; ^3^ Department of Ophthalmology, Massachusetts Eye and Ear Infirmary, Harvard Medical School, Boston, MA, USA; ^4^ Bascom Palmer Eye Institute, University of Miami Miller Medical School, Miami, FL, USA; ^5^ Department of Biological Sciences and Biotechnology, Tsinghua University, Beijing, China; ^6^ Windsor University School of Medicine, Brighton's Estate, Cayon, St. Kitts

**Keywords:** AMPK, ALK1, tumor angiogenesis

## Abstract

Anti-VEGF therapy has been proven to be effective in the treatment of pathological angiogenesis. However, therapy resistance often occurs, leading to development of alternative approaches. The present study examines if AMPK negatively regulates ALK1-mediated signaling events and associated angiogenesis. Thus, we treated human umbilical vein endothelial cells with metformin as well as other pharmacological AMPK activators and showed that activation of AMPK inhibited Smad1/5 phosphorylation and tube formation induced by BMP9. This event was mimicked by expression of the active mutant of AMPKα1 and prevented by the dominant negative AMPKα1. Metformin inhibition of BMP9 signaling is possibly mediated by upregulation of Smurf1, leading to degradation of ALK1. Furthermore, metformin suppressed BMP9-induced angiogenesis in mouse matrigel plug. In addition, laser photocoagulation was employed to evaluate the effect of metformin. The data revealed that metformin significantly reduced choroidal neovascularization to a level comparable to LDN212854, an ALK1 specific inhibitor. In conjunction, metformin diminished expression of ALK1 in endothelium of the lesion area. Collectively, our study for the first time demonstrates that AMPK inhibits ALK1 and associated angiogenesis/neovascularization. This may offer us a new avenue for the treatment of related diseases using clinically used pharmacological AMPK activators like metformin in combination with other strategies to enhance the treatment efficacy or in the case of anti-VEGF resistance.

## INTRODUCTION

Transforming growth factor-beta (TGF-β) family, including TGF-β, bone morphogenetic proteins (BMPs), and activins, play important roles in development of vascular system [1]. The proangiogenic effects of several members of this family are mediated via activin receptor-like kinase 1 (ALK1), a type I receptor [2], which has been shown to be essential for vascular development, remodeling and pathological angiogenesis. Although multiple BMPs can activate ALK1, BMP9 and BMP10 are specific activators in endothelial cells [3].

The expression of ALK1 overlaps with, although not limited to, sites of angiogenesis and vasculogenesis [4, 5]. In human, heterozygous mutations of ALK1 or endoglin (ENG), a non-kinase accessory protein for ALK1, account for hereditary hemorrhagic telangiectasia (HHT), a familial human vascular syndrome that is characterized by development of fragile and direct connection between arteries and veins, or arteriovenous malformations [6, 7]. The patients manifest cutaneous telangiectasias, increasingly severe nose bleeds, and gastrointestinal hemorrhage. In mouse, ablation of ALK1 in either endothelium or whole body causes death of embryos at midgestation due to severe vascular abnormalities [8, 9]. Loss of ALK1 in zebra fish impedes migration of cranial arterial endothelial cells along the direction of blood flow opposite to that of wild type, resulting in decreased endothelial cell number in arterial segments proximal to the heart and increased number distal to the heart [10].

ALK1 has also been found widely present on tumor blood vessels [11, 12] and endothelium of aorta in diabetic animal models that is upregulated by high glucose [13], suggesting a role in the pathogenesis of cancer and metabolic syndrome. Interestingly, *in vitro* studies have shown that ALK1 stimulates proliferation and migration of endothelial cells, whereas ALK5 inhibits these processes [14]. These studies provide an appealing rationale to target ALK1 as an anti-angiogenesis therapy [7]. In fact, two biosimilars that inhibit ALK1, an monoclonal antibody and ALK1-Fc (extracellular domain of ALK1 fused with Fc fragment), have shown inhibitory effects on angiogenesis in animal tumor model and are currently used in phase I clinical trials [2].

AMP-activated protein kinase (AMPK) has been well accepted as a therapeutic candidate for type 2 diabetes and obesity. In the last decade, AMPK has emerged as a metabolic tumor suppressor that plays a critical role in mediating the tumor suppressive function of LKB1 [15]. A plethora of research data have documented that AMPK regulates a broad spectrum of factors involved in cell metabolism, proliferation, survival, migration, and invasion [15, 16]. Interestingly, a retrospective investigation has revealed that the incidence of cancer is significantly reduced in patients with type 2 diabetes receiving treatment with metformin, an AMPK activator [17]. Therefore, AMPK appears to be an ideal target for both metabolic syndrome and cancer [15].

Previous studies have shown that AMPK participates in regulation of angiogenesis and function of endothelial cells [18, 19]. However, the precise role of AMPK in this aspect is rather controversial. While some studies point out the promoting effects on angiogenesis [20–24], others highlight an inhibitory role of AMPK that mediates the action of metformin [25–28]. The former is probably associated with stresses such as hypoxia and ischemia where activation of AMPK exerts a protective effect and promotes angiogenesis [29]. These two opposing effects are mediated by different mechanisms; on one hand, AMPK stimulates VEGF expression by activating HIF1α under hypoxia [24], and on the other hand, it inhibits mTOR, resulting in downregulation of VEGF [30]. Whether AMPK regulates angiogenesis via other mechanisms is completely unknown. The present study aims to examine if AMPK regulates ALK1 and associated angiogenesis.

## RESULTS

### AMPK inhibits ALK1-mediated signaling and tube formation

**Figure 1:**
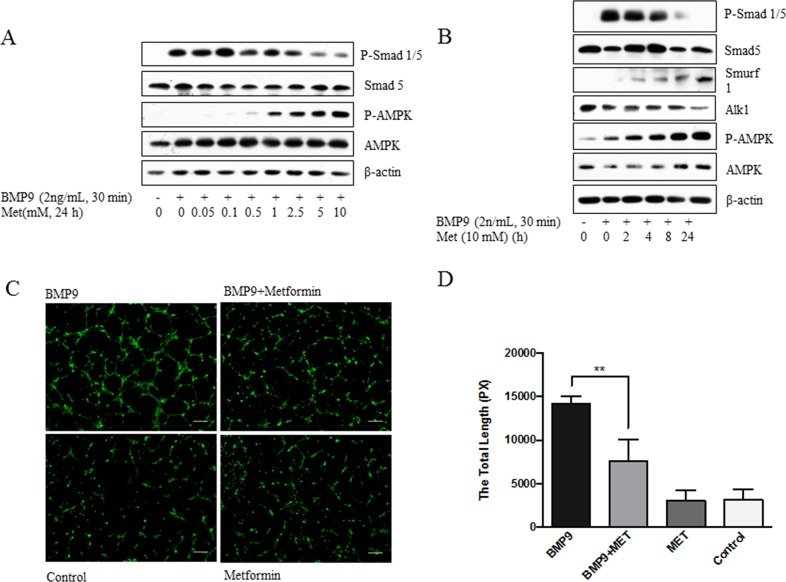
Inhibition of phosphorylated-Smad1/5 and tube formation by metformin **(A)** Dose-dependent effect. HUVECs were treated with metformin (Met) at varying doses, followed by BMP9. **(B)** Time-dependent effect. HUVECs were treated with metformin for different time (hours), followed by BMP9. Cell extracts (20 μg) were blotted with antibodies, as indicated in A and B. **(C)** Inhibition of tube formation. HUVECs were seeded on Matrigel and treated with or without BMP9 (10 ng/mL) and/or metformin (10 mM) for 7 hours. The cells were stained with Calcein AM and photos taken under fluorescent microscopy. Representative images are shown. Scale bar: 100 μm. **(D)** Quantitative analysis. Tube formation was calculated by lengths using ImageJ program and plotted. Analysis was performed from duplicate experiments, from which 5 visual areas were taken. Significance was tested by one way ANOVA (average pixels ± SD, n=10). ** *p*<0.01.

To determine if AMPK activation exerts an effect on ALK1-mediated angiogenesis, we examined the response of HUVECs to metformin by using phosphorylation of Smad1/5 and tube formation as readout. As shown in Figure 1A, with increasing doses of metformin, BMP9-evoked phosphorylation of Smad1/5 gradually decreased starting at 0.5 mM and reaching the maximum at 5 mM. At 10 mM, metformin inhibited Smad1/5 phosphorylation in a time-dependent fashion, giving rise to a maximal inhibition at 24-hour point (Figure 1B). Interestingly, the suppression of Smad phosphorylation correlated to upregulation of Smurf1 and downregulation of ALK1 (Figure 1B). Since Smurf1 is an E3 ubiquitin ligase, our results suggest that AMPK induces proteosomal degradation of ALK1 through the Smurf1-dependent mechanism. In parallel, we examined if metformin suppressed the ability of BMP9 to induce tube formation, a parameter of angiogenesis *in vitro*. The results revealed that BMP9 significantly stimulated tube formation derived from HUVCs, as compared to non-treatment control, while the effect of BMP9 was remarkably diminished by metformin (*p*<0.01), (Figure 1C–1D).

**Figure 2:**
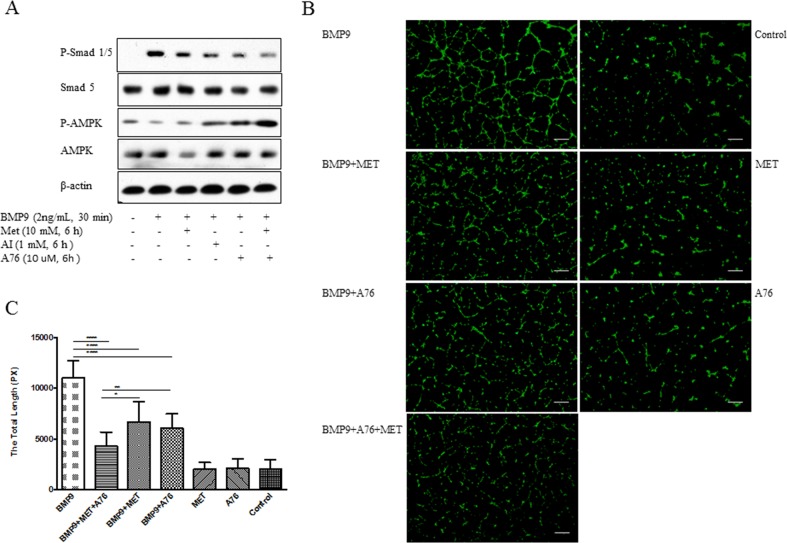
Inhibition of BMP9-induced phosphorylation of Smad1/5 and tube formation by different AMPK activators HUVECs were treated with metformin (Met), AICAR (AI), or A769662 (A76), followed by BMP9. Western blot was performed with antibodies, as indicated **(A)**. Tube formation **(B)** and statistical test (AMP activators-treated group vs BMP9 group) **(C)** were performed. Graph represents averages ± SD (n=10), * *p*<0.05, ** *p*<0.01, *** *p*<0.0001. Scale bar: 100 μm.

To ascertain if the effect of metformin could be reproduced by other AMPK activators, we used AICAR (AI) and A769662 (A76). As shown in Figure 2A, addition of each inhibited BMP9-induced phosphorylation of Smad1/5 to the similar extent as did metformin (Figure 2A). Similarly, tube formation was correspondingly suppressed (Figure 2B). Combination of metformin and A769662 gave more inhibition than each alone (Figure 2).

**Figure 3:**
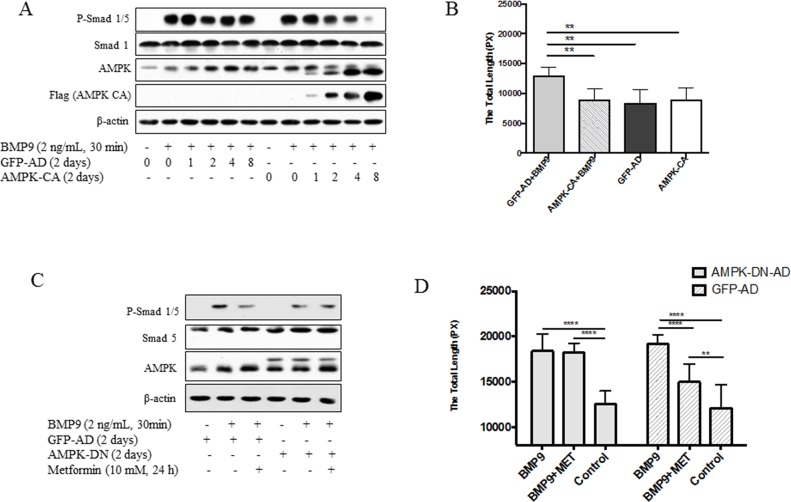
Effects of AMPK mutants on Smad1/5 phosphorylation and tube formation **(A)-(B)** HUVECs were infected with adenovirus encoding active mutant of AMPKα1 (AMPK-CA) or GFP in different volumes (μl) for 2 days and then treated with BMP9. (A) Cell extracts were blotted with antibodies, as indicated. (B) Tube formation assay. HUVECs were infected with adenovirus for AMPK-CA or GFP for 2 days, and tube formation was performed, as described in Figure 1. Statistical analysis of tube formation was performed. Graph represents averages ± SD (n=10). **p<0.01. **(C)-(D)** HUVECs were infected with adenovirus expressing the dominant negative mutant of AMPKα1 (AMPK-DN) or GFP. (C) The cells were treated with or without metformin, followed by BMP9 and cell extracts blotted with antibodies, as indicated. (D) Tube formation was assayed on HUVECs infected with AMPK-DN or GFP adenovirus and treated with or without BMP9 (10 ng/mL) and/or metformin (10 mM) for 6 hours. Graph represents averages ± SD of tube length (n=10). Two way ANOVA was used to test statistical significance. **p<0.01, *** *p*<0.0001.

Next, we expressed the active mutant of AMPKα1 subunit in HUVECs by using an adenoviral vector Ad-AMPK-CA and examined the response of the cells to BMP9. As shown in Figure 3A-3B, with increasing doses of Ad-AMPK-CA, the ability of BMP9 to stimulate phosphorylation of Smad1/5 and tube formation was diminished, whereas the control vector Ad-GFP that expresses GFP had no such effect. Furthermore, in HUVECs infected with Ad-GFP, metformin was effective in inhibiting BMP9-induced phosphorylation of Smad1/5 and tube formation. When the dominant negative mutant of AMPK (Ad-AMPK-DN) was infected, the effect of metformin was blunted, as compared to GFP adenoviral control (Figure 3C–3D).

**Figure 4:**
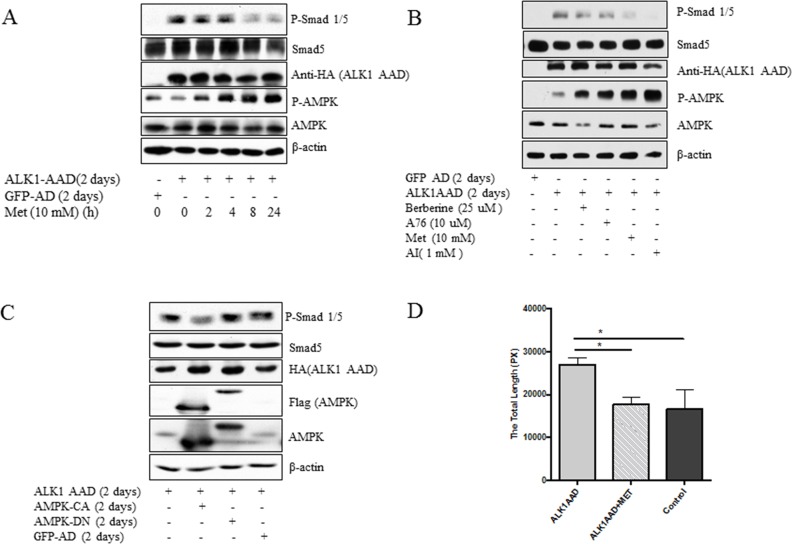
Inhibition of active mutant of ALK1 by AMPK **(A)** HUVECs were infected with ALK1AAD or GFP adenovirus for 2 days and treated with metformin (10 mM) for 2 to 24 hours. **(B)** HUVECs were infected with adenovirus expressing an active mutant of ALK1 (ALK1AAD) or GFP adenovirus for 2 days and then treated with metformin (Met), berberine (25 μM), AICAR (AI), or and A769662 (A76) overnight. **(C)** Adenovirus expressing ALK1AAD was co-infected with adenovirus expressing AMPK-CA, AMPK-DN, or GFP. Western blot was performed with antibodies, as indicated in A-C. **(D)** HUVCs were infected with ALKAAD adenovirus and tube formation was conducted with or without metformin (10 mM) for 6 hours and graph plotted. Adenovirus expressing GFP served as a control. Statistical analysis of tube formation in C was performed as described in Figure 1. Graph represents averages ± SD (n=5). *p<0.05.

To test if the activated ALK1 exhibits the same effect as BMP9, ALK1AAD, a constitutively active mutant was delivered to HUVECs by the adenoviral vector. The results demonstrated that ALK1AAD had the same effects as did BMP9 and that the treatment of the cells with metformin (Figure 4A) and other activators (AI, A76, berberine) (Figure 4B) inhibited the ability of the active ALK1 to stimulate Smad1/5 phosphorylation. Likewise, the stimulating effect of active ALK1 was blunted by the active mutant of AMPK, but not by the dominant negative mutant of AMPK (Figure 4C). Finally, metformin also suppressed tube formation induced by the active ALK1 mutant (Figure 4D).

### Metformin inhibits BMP9-induced angiogenesis *in vivo*

**Figure 5:**
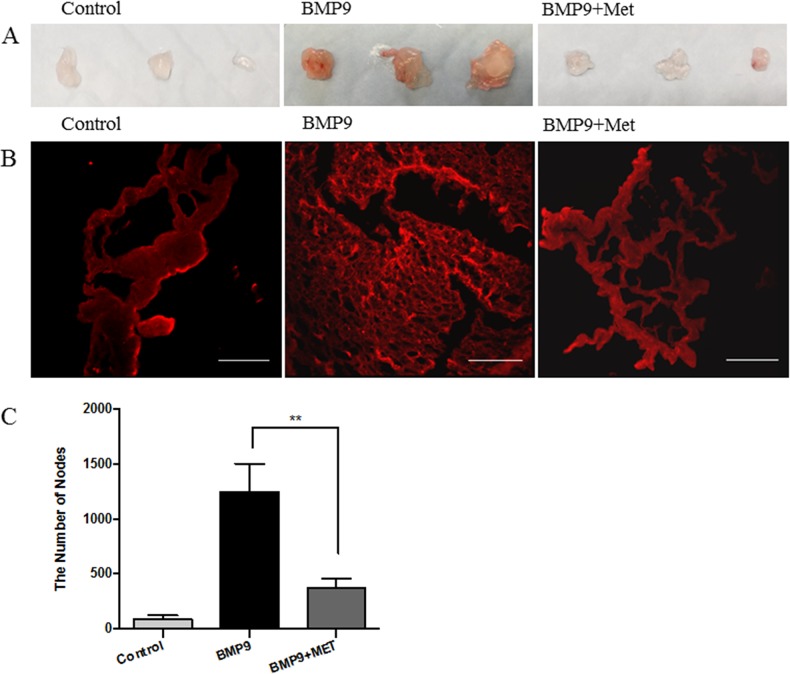
Metformin inhibits angiogenesis on mouse Matrigel plug Matrigel was subcutaneously injected into the back of mice in the presence or absence of BMP9 (10 ng/mL). The mice were administered intraperitoneally with metformin (150 mg/kg/day) and/or PBS or 7 days. Matrigel plugs were then excised, sectioned and analyzed. **(A)** Gross anatomy of freshly removed Matrigel plug. **(B)** Slides were incubated with Dy594-lectin and images taken under fluorescent microscope. Scale bar: 100 μm. **(C)** The dense capillary structures were analyzed by nodes using ImageJ program and plotted. Graph represents averages of five different sights ± SD (n=5). Significance was tested by one way ANOVA, ** *p*<0.01.

To assess the role of AMPK activation in angiogenesis, we first conducted the Matrigel plug assay, where BMP9 was mixed with growth factor-reduced Matrigel and injected subcutaneously to the back of mice, followed by intraperitoneal (i.p.) administration of metformin (150 mg/kg/day) up to 6 days. The plugs were exercised, sectioned and stained with Dy594-lectin. As shown in Figure 5, plugs with BMP9 were larger and red, whereas those with BMP6 and metformin were pale and smaller, similar to the control plugs without BMP9. Dense capillary structure was observed in sections of the BMP9 plugs, but which was significantly less in the presence of metformin (Figure 5B–5C). This result strongly suggests that AMPK inhibits angiogenesis evoked by the activation of BMP9-ALK1 signaling cascade.

### Metformin inhibits laser-induced choroidal neovascularization

**Figure 6:**
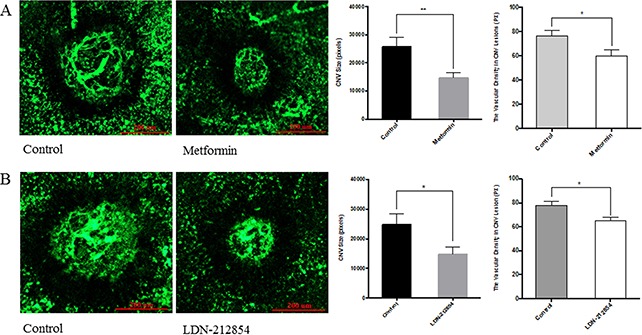
The effect of metformin on laser-induced choroidal neovascularization (CNV) in mice C57BL/6J mice were used for laser-induced CNV model. Four lesions were induced using the laser photocoagulation after anesthesia with ketamine hydrochloride. **(A)** Animals were equally divided into two groups (8 mice/group), treated with PBS or metformin (150 mg/kg/day) *i.p*. once daily from the day prior to laser irradiation (Day-1) to Day 6. For the evaluation of CNV size, at Day 7, mice were deeply anesthetized and perfused with FITC-lectin. The mice were sacrificed and the eyes were enucleated and fixed in 4% paraformaldehyde. RPE-choroid tissue was flat-mounted and observed under fluorescein microscope. Size of CNV was measured by μm^2^ and vascular density in CNV lesion by ImageJ program. Significance was tested by student t test (average pixels ± SEM, n=7). *p<0.05, ** *p*<0.01. **(B)** Animals were injected *i.p*. with PBS or LDN-212854 (6 mg/kg, twice daily) and processed as described for A. CNV size and vascular density in CNV lesions were determined. Scale bar: 200 μm.

We next employed the laser-induced choroidal neovascularization (CNV), a model of exudative AMD, to assess the effect of AMPK. Animals were injected intraperitoneally with metformin or PBS as control once daily from one day (Day 1) before laser treatment to Day 6 post treatment. At the endpoint (Day 6), mice were deeply anesthetized and perfused with FITC-lectin. RPE-choroid preparations were flat mounted and examined by fluorescence microscopy. CNV size in eyes from metformin treated group was significantly smaller than the PBS treated controls (*p*<0.01, Figure 6) and vascular density in the CNV lesion was also less (*p*<0.05). In another set of experiment, animals were treated by intraperitoneal injection of PBS or LDN-212854 (6 mg/kg, twice daily), an ALK1/2 specific inhibitor. LDN-212854 also significantly inhibited laser-induced CNV and vascular density (*p*<0.05).

**Figure 7:**
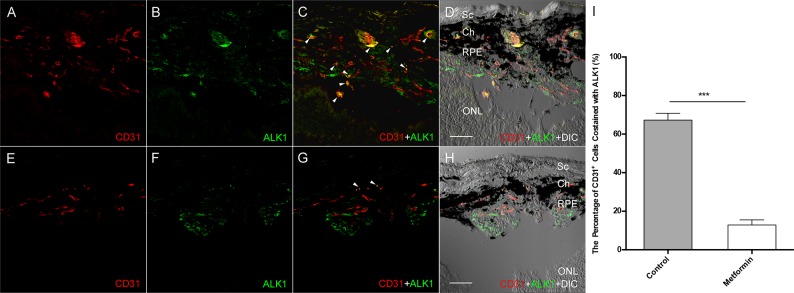
The effect of Metformin on ALK1 expression in Laser-induced CNV Lesion was created as described in Figure 6A, except that eyes were harvested at day 5. The cryosectioned slides were incubated with antibodies against ALK1 and CD31, an endothelium marker, and DAPI Extensive staining of CD31 (red) and ALK1 (green) is seen in control eyes from animals treated with PBS **(A** and **B)**. Many CD31 positive cells are also positive for ALK1 staining, indicating they are endothelial cells in the new blood vessels that expressed ALK1 **(C, arrowheads)**. A DIC (differential interference contrast) image was superimposed to the fluorescent images to provide structural information **(D)**. In contrast, The CD31 and ALK1 staining is much less in the eyes from metformin treated animals **(E** and **F)**, and almost no co-localization was present **(G** and **H)**. **(I)** Co-stained signal of CD31 and ALK1 was compared in the presence and absence of metformin and plotted. Graph represents averages ± SD from three independent experiments. ***p<0.001. Scale bars: 50 μm. Sc: Sclera; Ch: Choroid; RPE: Retinal pigment epithelium; ONL: outer nuclear layer.

To assess if metformin treatment downregulates the expression of ALK1 during the course of CNV induction, we examined the ALK1 expression and localization in eyes after laser treatment. In this experiment, the mice were sacrificed at day 5 and RPE-choroid slides were double labeled with antibodies against CD31 and ALK1. As shown in Figure 7, the expression of ALK1 (green) was strongly co-localized with CD31 (Fig. 7C, arrowheads) in many cells of the laser lesion area (Figure 7A to D). In contrast, less ALK1 expression was seen in animals treated with metformin. The CD31 staining was also much less in metformin treated animals (Figure 7E to F). Furthermore, there was very few co-localization of ALK1 with CD31 in metformin treated animals (Figure 7G, arrowheads), indicating very low expression of ALK1 expression in endothelial cells in the laser lesion after metformin treatment (*p*<0.001, Figure 7I). The *in vivo* action of metformin on ALK1 abundance holds the same as that *in vitro* (Figure 1). In contrast, we did not detect a significant difference in TGF-β in the PBS and metformin groups (Data not shown).

## DISCUSSION

Dysregulation of BMP-signaling has been recently indicated to be involved in cancer and diabetic vascular complications [2, 13]. A prominent outcome of the dysregulated BMP-signaling is the increased angiogenesis and vascularization. ALK1 has been characterized as a critical player in these processes. Hence, a great deal of effort has been vested on developing therapies targeting ALK1 and the associated pathway [2]. In fact, several drugs, including biosimilars and chemical compounds, are currently at phase I clinical trial stage. In the present study, we explored if AMPK serves as a candidate for the inhibition of the ALK1-mediated signaling events and associated angiogenesis. Our results showed that metformin as well as other pharmacological activators of AMPK attenuated Smad1/5 phosphorylation and tube formation in HUVECs in response to BMP9, an event that was mimicked by an active mutant of AMPK and blunted by its dominant negative mutant. Overexpression of constitutive active mutant of ALK1 enhanced phosphorylation of Smad1/5, which was diminished by metformin or expression of active AMPK. Likewise, stimulation of tube formation by the active mutant of ALK1 was abolished by metformin. Additionally, the Matrigel plug analysis demonstrated that treatment of mice systemically with metformin remarkably suppressed vascularization induced by BMP9. Furthermore, we employed a laser-induced CNV assay to corroborate the role of metformin in prevention of vascularization. Our data revealed that metformin significantly reduced laser-induced CNV sizes and vascular density in the CNV lesions, which was comparable to the ALK1/2 inhibitor LDN212854. Consistently, metformin decreased ALK1 abundance around CNV areas. Altogether, the present study provides compelling evidence that AMPK inhibits BMP9/ALK1-mediated angiogenesis/vascularization. Hence, our findings offer new insight into therapies for associated diseases.

Blood vessel formation is a complex process involving many factors, among which ALK1 plays a pivotal role. However, many reports on the action of ALK1 signaling in endothelial cells have revealed paradoxical results. Although majority of literature supports a promoting role of ALK1 in angiogenesis, several studies have shown opposite effects [31–35]. Possible explanations for these opposing results may rely on the context-specific nature of ALK1 signaling events, dosage and length of activation, and interaction with other proangiogenic factors as well as cell type and culture conditions. For example, ALK1 has been shown to inhibit proangiogenic effect of bFGF and VEGF [36]. Secondly, it has been shown that Notch cooperates with BMP9/ALK1 to allow stalk cell phenotype and inhibit VEGF signaling, tip cell formation and endothelial sprouting, so as to maintain balance of angiogenesis [33, 37]. It appears that formation of mature blood vessels requires concert action of multiple factors. Dysregulation of one, which often occurs under pathological circumstances such as cancer and diabetic retinopathy, may cause disturbance of the other, resulting in suppression or overactivation. Such an alteration could be corrected by addition of one or the other [35]. An obvious example is that heterozygous mutation of ALK1 or ENG causes HHT characterized by arteriovenous malformations and hypervascularization, which concurs with increased VEGF signaling. Therefore, anti-VEGF therapy can be considered to restore angiogenic-angiostatic balance in HHT disease [37, 38].

We are aware of a recent study showing the negative effect of BMP9/ALK1 on neovascularization in mouse models using CNV, where upregulation of BMP9/ALK1 signaling was observed [35]. Adenovirus-delivered BMP9 inhibited neovessel formation. This finding is surprisingly different from ours. At present, we could not interpret the discrepancy. Nevertheless, we have presented both *in vitro* and *in vivo* data to support the pro-angiogenic effect of ALK1.

It has been known that VEGF plays an import role in pathological angiogenesis including cancer, neovascular AMD and proliferative diabetic retinopathy and thus it is a well-received therapeutic target [39–41]. In fact, antibody therapy against VEGF and VEGFR-Fc trap have exhibited considerable therapeutic effects. However, therapeutic resistance and side effects are not neglectable [42]. Therefore, alternative approach ought to be sought. Altered expression of ALK1 has been found to be a pathological factor in cancer as because of dysregulated angiogenesis [2]. In keeping with this, a recent gene expression study on breast cancer specimens has defined expression of ALK1 in endothelium as a specific prognostic factor for metastasis [12]. An early study using animal model suggests that ALK1 plays an important role in therapeutic resistance to anti-VEGF therapy [11]. Therefore, blocking ALK1 by specific antibody or extracellular segment of ALK1 fused with Fc fragment of IgG (ALK1-Fc) has been employed in animal models to suppress angiogenesis, leading to inhibition of tumor growth and metastasis. Several reports have documented that targeting ALK1 is effective in monotherapy or combination with anti-VEGF or other therapies [2, 7].

Previous studies have shown that metformin could inhibit VEGF-mediated pathological angiogenesis [25–28]. Our present study delineates an additional mechanism by which it inhibits angiogenesis via inhibition of ALK1. Our data have revealed that metformin upregulates Smurf1, an E3 ubiquitin ligase, suggesting that this may lead to degradation of ALK1. A recent study of Wei et al demonstrated that AMPK could phosphorylate Smurf1 and increased its activity [43]. In our study, we could not detect phosphorylation of Smurf1 by AMPK, but rather observed an increase in Smurf1 abundance, possibly resulting from increased stability. In a separate study, we have found that knockdown of Smurf1 with siRNA prevents the degradation of ALK2 in fibroblast cells. Thus, we believe that downregulation of ALK1 occurs via the same mechanism.

Of note, CD31 and ALK1 were not completely co-localized in the study. This is a little surprise to us, as previous studies reported that ALK1 is predominantly expressed in endothelial cells. One interpretation is that it is also present in scar tissue of the lesion. Alternatively, increased expression of ALK1 in new blood vessels might occur and be able to be captured within certain time window (e.g. an early event) of CNV induction. Nonetheless, our results demonstrated that co-localization of fluorescence-labeled CD31 and ALK1 was remarkably less in the metformin-treated samples than that of control. Furthermore, both metformin and LDN212854 suppressed CNV size, suggesting that ALK1 plays an important role in the pathological process of CNV.

In conclusion, our study for the first time demonstrates that AMPK activation inhibits ALK1 and associated angiogenesis/neovascularization. This offers us a new avenue for the treatment of disorders associated with upregulation of BMP/ALK1 signaling events, which is especially important for pathological angiogenesis. Thus, metformin can be used in combination with other strategies to enhance the treatment efficacy or in the case of anti-VEGF resistance. Metformin has been used for many years in clinics with an excellent safety record. Our work provides experimental evidence of its anti-angiogenetic potentials. Re-tasking metformin as well as other AMPK activators used in human including aspirin, berberine, and resveratrol, as anti-angiogenesis therapeutic drugs, either as a monotherapy or in combination with other therapies could provide a safe and affordable alternative for patients.

## MATERIALS AND METHODS

### Reagents

Monoclonal antibodies against p-AMPKα (Thr172), total AMPK α, p-Smad1/5 and total-Smad1 or 5 were purchased from the Cell Signaling Technologies Company (Beverly, MA). Metformin, LDN212854, 5-Aminoimidazole-4-carboxamide ribonucleotide (AICAR), and monoclonal antibodies for β-actin and flag were from Sigma (St Louis, MO). Antibodies for ALK1 and TGF-β were from Abcam (Cambridge, MA). Recombinant mouse BMP9 was from Biolegend (San Diego, CA). Growth factor-reduced Matrigel and antibody against CD-31 was from BD Biosciences (San Jose, CA). Optimal cutting temperature medium (OCT) was from Sakura Finetek USA Inc (Torrance, CA). DyLight 594 labeled Griffonia Simplicifolia Lectin (GSLI)isolectin B4 (Dy594-lectin) and FITC-lectin were from Vector Labs (Burlingame, CA). Ketamine hydrochloride and xylazine were from Phoenix Pharmaceutical, Inc (St. Joseph, MO). Phenylephrine and tropicamide were from Alcon (Humacao, Puerto Rico).

### Animals

Animal use for Matrigel plug angiogenesis study was approved by Institutional Animal Care and Use Committee (ACUC) of Boston University (animal protocol: AN-14726) and for laser coagulation assay approved by IACUC of Massachusetts Eye and Ear Infirmary (animal protocol: 15-015 MEEI). C57BL/6J mice (6~8 weeks old) were purchased from Charles River Laboratories (Shrewsbury, MA).

### Cell culture

Human Umbilical Vein Endothelial Cells (HUVECs) were purchased from the American Type Culture Collection (ATCC) (Rockville, MD, USA). HUVECs were maintained in EBM-2 Basal Medium purchased from Fisher Scientific (manufactured by Lonza), supplemented with EGM-2 BulletKit (Lonza) and penicillin/streptomycin at 37°C and 5% CO_2_. Two batches of HUVECs were used and each was subcultured less than 10 passages. AAV293 purchased from Agilent Technologies (Santa Clara, CA) were cultured with DMEM medium supplemented with 10% fetal bovine serum and penicillin/streptomycin at 37°C and 5% CO_2_.

### Western blot

Cells were lysed in lysis buffer [44] and cell debris was removed by centrifugation at 14,000xg at 4°C for 15 min. Supernatant extracts were quantified using Bio-Rad protein assay kit. Equal amounts of protein (20-40 μg) were subjected to SDS-PAGE and transferred to PVDF membranes. After transfer, the membranes were incubated with blocking solution and sequentially blotted with primary and second antibodies. The blots were finally developed by the enhanced ECL kit (Thermo Fisher Scientific).

### Preparation of adenovirus

cDNAs for the active mutant of human AMPKα1 subunit with deletion of amino acids 313 to 390 and for the dominant negative mutant (D159A) were subcloned to pAdTrack-CMV vector [45]. The constitutively active mutant of ALK1 (ALK1AAD) [46], tagged with hemagglutinin (HA) epitope at the carboxyterminus were subcloned to pAdTrack-CMV vector. Recombinant viral genomes were obtained using the bacteria BJ5183 and transfected into AAV-293 cells by the standard calcium phosphate precipitation method. Adenoviruses were amplified and purified using the AdEasy virus purification kit (Agilent Technologies).

### Tube formation assay

Matrigel-based tube formation [47] was used to evaluate the ability of endothelial cells to form capillary-like structures. In brief, 24-well plates were coated with Growth Factor-Reduced Basement Membrane Matrix (50 μL/cm^2^) at 37°C for 30 minutes. Upon Matrigel solidification, HUVECs (42,000 viable cells/cm^2^) were seeded onto each well, and cultured in non-supplemented EMB2 in the presence or absence of chemical compounds, as indicated in figure legends, for 6-8 hours. In the case of adenovirus infection, the cells were infected 48 fours prior to seeding onto Matrigel. At the end of tube formation test, Calcein AM (2 μg/mL) was added to the cell culture medium and the cells incubated for 20 minutes. Images were taken under a fluorescent microscope and tube formations were quantified using ImageJ program (developed by Wayne Rasband, National Institutes of Health, Bethesda, MD; available at http://rsb.info.nih.gov/ij/index.html).

### Matrigel plug assay

The assay was conducted as described previously [47]. C57BL/6J mice at 6 weeks of age were purchased from Charles River Laboratories and divided into three groups, PBS, BMP9, and BMP9 with metformin treatment, each containing 4 mice. Growth factor–reduced Matrigel (19.49 mg/mL protein) was mixed at 4°C with equal volume of mixture of heparin (50 μg/mL) and/or BMP9 (10 ng/mL) in PBS and injected subcutaneously (0.3 ml) to the left and right side of back midline on mice after inhaling anesthesia with isoflurane. Matrigel with PBS and heparin was used as a control group. For the treatment group, metformin (150 mg/kg/day) or PBS vehicle was injected intraperitoneally once a day from day 1 to day 6. Seven days after injection, mice were sacrificed by *i.p*. injection of pentobarbital (250 mg/kg), followed by cervical dislocation and Matrigel plugs were harvested and immersed in OCT medium. Serial sections of 20-micron thickness were cut using a cryostat and stored at -80°C. Vessel formation was analyzed by histological staining using Cy5-labeled lectin (Thermo Fisher Scientific).

### Laser-induced choroidal neovascularization (CNV) model

C57BL/6J mice (male, 8 weeks old, 25–30 g) were used for laser-induced CNV animal model according to the protocol described by Giani et al [48]. Mice were anesthetized by intraperitoneal injection of ketamine hydrochloride (50 mg/kg and xylazine (10 mg/kg). Pupils were dilated with topical 5% phenylephrine and 0.5% tropicamide before induction of CNV by laser photocoagulation (Oculight GLx Laser System, IRIDEX Corporation, Mountain View, CA). The induction was conducted in the following settings: a 532-nm laser, 100 mW power, 100 μm spot size and 0.1 second duration. Lesions were induced at the 3, 6, 9, and 12 o’clock meridians centered on the optic nerve and located two or three disc diameters. The disruption of Bruch's membrane was confirmed by immediate bubble formation after laser photocoagulation.

For CNV size assay, mice (8 mice/group) were divided into the following groups: PBS vehicle vs metformin; PBS vs LDN212854. They were injected intraperitoneally with PBS or metformin (150 mg/kg/day) once daily from the day before the laser induction to day 6. LDN212854 at the dose suggested by manufacturer (6 mg/kg) was injected *i.p*. twice daily from the day before the laser induction to day 6. For evaluation of CNV size, on Day 7, mice were deeply anesthetized, perfused with FITC-lectin (Vector Laboratories), and sacrificed by cervical dislocation. Eyes were enucleated and fixed in 4% paraformaldehyde. The RPE-choroid tissue was flat mounted and observed under fluorescein microscope. ImageJ software was used to delineate and measure the area of the CNV in each image. For immune staining with TGF-β and ALK1, mice were sacrificed and the eyes were enucleated on Day 5.

### Immunofluorescence

At the indicated time points (Day 5) after CNV induction, eyes were enucleated, fixed in 4% paraformaldehyde, and embedded in OCT. During enucleating and embedding procedure, the spatial orientation of the eye was preserved to identify CNV lesions of particular interest. Serial sections of 10-micron thickness were cut using a cryostat and stored at -80°C. Slides were dry out at 37°C for 15 min and fixed in ice-cold methanol for 15 min, and then blocked with blocking buffer (0.5% bovine serum albumin and 0.3% Triton in PBS) for 1 h at room temperature. The slides were incubated with rabbit monoclonal antibody for ALK1 and rat antibody for CD31 antibody overnight at 4°C, and then with goat anti-rabbit, goat anti-rat IgG antibodies and DAPI for 1 hours at 37°C. After washing with PBS, slides were visualized under confocal laser-scanning microscope (FluoView FV-1000; Olympus).

### Statistical analysis

The data are expressed as mean standard error of the mean (SEM) or standard deviation (SD). Significance among two groups was determined by student t test and multiple groups using one way ANOVA or two way ANOVA, followed by post hoc Tukey test for multiple comparisons.

## Abbreviations

AICAR: 5-Aminoimidazole-4-carboxamide ribo-nucleotide
AMPK: 3′ adenosine monophosphate-activated protein kinase
ALK1: Activin receptor-like kinase
BMP: bone morphogenetic protein;
DAPI: 4′,6-diamidino-2-phenylindole
ENG: endoglin
HHT: hereditary hemorrhagic telangiectasia
HUVEC: human umbilical vein endothelial cell
CNV: choroidal neovascularization
Smurf1: SMAD-specific E3 ubiquitin ligase 1
TGF-β: transforming growth factor beta
VEGF: Vascular endothelial growth factor
